# MicroRNAs miR-27a and miR-143 Regulate Porcine Adipocyte Lipid Metabolism

**DOI:** 10.3390/ijms12117950

**Published:** 2011-11-15

**Authors:** Tao Wang, Mingzhou Li, Jiuqiang Guan, Penghao Li, Huiyu Wang, Yanqin Guo, Surong Shuai, Xuewei Li

**Affiliations:** Institute of Animal Genetics & Breeding, College of Animal Science & Technology, Sichuan Agricultural University, Ya’an, Sichuan 625014, China; E-Mails: wanttao@gmail.com (T.W.); mingzhou.li@163.com (M.L.); 79184122@qq.com (J.G.); 45085733@qq.com (P.L.); 253620495@qq.com (H.W.); 610810793@qq.com (Y.G.)

**Keywords:** porcine, adipogenesis, adipolysis, miR-27a, miR-143

## Abstract

MicroRNAs (miRNAs) are non-coding small RNAs that play roles in regulating gene expression. Some miRNAs have been classed as epigenetic regulators of metabolism and energy homeostasis. Previous reports indicated that the miRNAs miR-27a and miR-143 were involved in lipid metabolism in human and rodents. To determine the roles of porcine miR-27a and miR-143 in adipocyte lipid metabolism, porcine adipocytes were cultured and allowed to induce differentiation for 10 days. The lipid-filled adipocytes were then transfected with miRNA mimics and inhibitors. We measured how the indicators of adipogenesis and adipolysis in porcine adipocytes were affected by the over-expression and by the inhibition of both miR-27a and miR-143. The results indicated that the over-expression of miR-27a could accelerate adipolysis releasing significantly more glycerol and free fatty acids than the negative control (*P* < 0.001), while the mimic of miR-143 expression, promoted adipogenesis by accumulating more triglycerides (*P* < 0.001) in the adipocytes. In addition, we demonstrated that there was good correlation (*r* > 0.98, *P* < 0.001) between the indicators of adipolysis in cell lysates and in the culture medium.

## 1. Introduction

MicroRNAs (miRNAs), the endogenous small (~22 nt) non-coding RNAs, are generally regarded as negative regulators of gene expression that inhibit translation of mRNA by binding to the 3′ untranslated region (3′ UTR) of target mRNAs in animals [1,2]. A role for miRNAs in lipid metabolism was first described in *Drosophila melanogaster*, where the loss of miR-14 increases body fat content [3]. In vertebrates, the first miRNA linked to metabolism was the pancreatic-islet-enriched miR-375 that interferes with insulin secretion [4]. Recently, several miRNAs, including miR-27a and miR-143, have been shown to regulate lipid metabolism in human and rodent cell models [5,6]. In mouse 3T3-L1 cells, the level of miR-27a expression gradually decreased upon adipogenesis, and the ectopic expression of miR-27a in 3T3-L1 preadipocytes suggested that miR-27a might suppress adipocyte differentiation by repressing the expression of *PPAR*γ [7]. Elsewhere, it has been reported that miR-143 is up-regulated after the induction of differentiation in human preadipocytes and in mouse 3T3-L1 cells [8,9]. The introduction of antisense oligonucleotides against miR-143 in preadipocytes was reported to inhibit their differentiation by down-regulating *ERK5*, suggesting that miR-143 plays a role in accelerating adipocyte differentiation [8]. The importance of miRNAs in regulating lipid metabolism has only recently been discovered, but this is likely to become a burgeoning area of investigation in the future.

Adipose tissue, the major storage site of triglycerides (TGs), is an important fuel source that provides free fatty acids (FFA) and gluconeogenic carbons in the form of glycerol. Adipose tissue is a critical organ, made even more important by the escalating appearance of obesity and diabetes in many populations [10]. Therefore, insights into the role of miRNAs in the regulation of lipid metabolism may contribute to our understanding of these diseases [11,12].

The pig is an attractive biomedical model for human health, including for obesity [13] and diabetes [14]. In the present study, we used synthetic miRNA mimics and inhibitors in gain- and loss-of-function experiments to investigate the roles of miR-27a and miR-143 in lipid metabolism in porcine adipocytes.

## 2. Results and Discussion

### 2.1. Identification of Porcine Adipocyte

**Figure 1:**
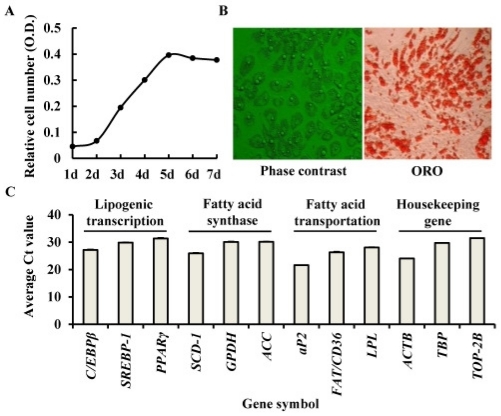
Adipocyte differentiation of S-V cells isolated from porcine subcutaneous adipose tissue. Confluent preadipocytes were exposed to a differentiation cocktail (insulin, 3-isobutyl-1-methylxanthine, dexamethasone) for lipid accumulation. (**A**) The growth curve of the preadipocytes; (**B**) Phase contrast images of terminal differentiation adipocytes obtained 10 days after hormonal induction (left) and visualized by Oil Red O (ORO) staining (**right**); lipid droplets were stained bright red (×100); (**C**) The mRNA abundance of nine adipocyte-specific marker genes in porcine adipocytes after 10 days of differentiation. Values are mean ± S.D. The full names of the genes are listed in the footnote to Table 1.

The porcine S-V cells derived from subcutaneous adipose tissue were placed in culture flasks for clone expansion. As shown in the Figure 1(A), the proliferation continued from days 1 to 5 till confluence of the cells. Differentiation was initiated at day 7 and terminated at day 17 (Figure 1(B)). The detectable mRNA expression of nine adipocyte-specific marker genes in the terminal differentiation adipocytes (Figure 1(C)) and evidence of the morphological characteristics after ORO staining (Figure 1(B), right), confirmed that the porcine preadipocytes had differentiated to *bona fide* adipocytes.

### 2.2. The Establishment of a miRNA Transfection System for Porcine Adipocytes

**Figure 2:**
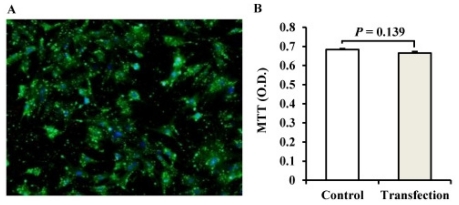
Transfection of FAM-labeled (green) delivery into porcine adipocytes. (**A**) A merge image was obtained (FAM/DAPI) for monitoring the transfection efficiency; the nucleus stained blue with DAPI for fluorescence microscopy; (**B**) The transfection cytotoxicity was determined using the MTT test. The Student’s *t*-test (two-tailed) was used for analysis of the results (*n* = 3). Values are mean ± S.D.

We obtained high transfection efficiency (~90% Figure 2(A)) as measured by the uptake of the FAM-labeled delivery control at a concentration of 100 nM in Lipofectamine 2000 (2:1, v/v). The transfected cells continued to exhibit normal viability when compared with the control groups (*P* = 0.139, Figure 2(B)). These results show that lipid-mediated miRNA transfection of fully differentiated porcine adipocytes took place high efficiency and with no detectable cytotoxicity, making them suitable for use in the subsequent analysis.

### 2.3. The Roles of miR-27a and miR-143 in Porcine Adipocyte Lipid Metabolism

**Figure 3:**
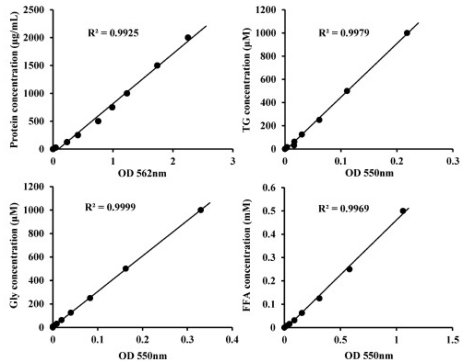
The standard curves of protein, TG, glycerol and FFA were constructed by using colorimetric method.

To investigate the potential functions of miRNAs in the lipid metabolism of porcine adipocytes, we performed over-expression and knockdown experiments by direct transfection of short double-stranded RNAs (miRNA mimics) and their OMe-modified antisense oligonucleotides (miRNA inhibitors). We next investigated the influence of miRNA on phenotypes of pig mature adipocytes via adipogenesis (deposition of TG) and adipolysis (TG are broken down to glycerol and FFA). Four high confidence standard curves (*R^2^* > 0.99, Figure 3) were obtained for concentration calculation.

**Figure 4:**
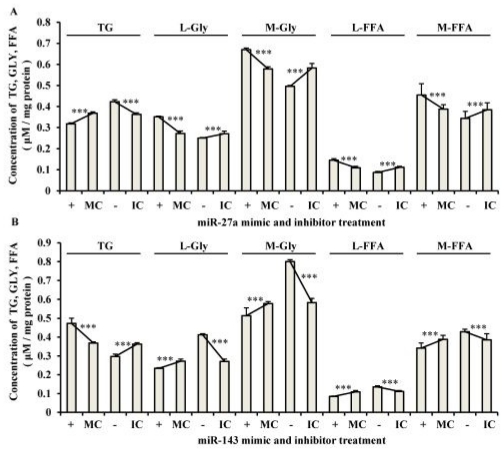
Analysis of lipid metabolism in adipocytes transfected with mimics and inhibitors of the miRNAs. (**A**) miR-27a; (**B**) miR-143. MC and IC represent the mimic and inhibitor controls, respectively. “+” and “−” indicate the up- and down- regulation of the expression of the specific miRNA; respectively. *** *P* < 0.001, Student *t*-test. L-Gly: lysate Gly release; M-Gly: medium Gly release; L-FFA: lysate FFA release; M-FFA: medium FFA release.

As shown in Figure 4(A), a lower TG concentration in cell lysates (*P* < 0.001), and higher glycerol and FFA concentrations both in cell lysates and in culture medium (*P* < 0.001) were found in miR-27a mimic group compared with the concentrations in the negative control. As expected, the opposite results were observed when miR-27a inhibitor groups were compared with the control group. For the miR-143 mimic group (Figure 4(B)), in contrast to the results for miR-27a, a higher TG concentration in cell lysates (*P* < 0.001) and lower glycerol and FFA concentrations both in cell lysates and in the culture medium (*P* < 0.001) were found when this group was compared with the negative control. In addition, the results observed in the miR-143 inhibitor groups were the opposite of those for the mimic group.

**Figure 5:**
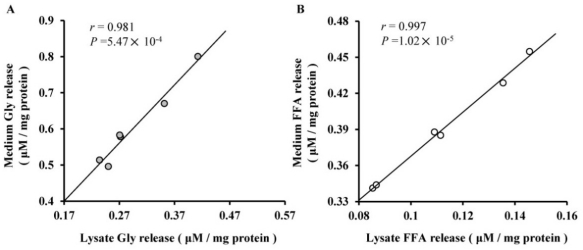
Pearson’s correlation of the concentrations of glycerol and FFA between the cell lysates and the culture medium. (**A**) Glycerol (Gly) concentrations; (**B**) Free fatty acid (FFA) concentrations. The concentrations were normalized to the protein content (μM/mg protein) using a bicinchoninic acid (BCA) assay kit.

These results indicated that miR-27a suppressed adipocyte differentiation, while miR-143 promoted adipocyte differentiation, confirming previous reports in human and rodent [7,8]. During adipogenesis, the abundance of miR-27a was inversely correlated with that of adipogenic marker genes, such as *PPAR*γ and adiponectin, and the abundance of miR-27a in the mature adipocyte fraction of obese mice was down-regulated compared with that of the leaner mice [7]. It has been reported that miR-143 is up-regulated after the induction of differentiation in human preadipocytes and in mouse 3T3-L1 cells [8,9], and the higher abundance of miR-143 has also been detected in the mesenteric fat of high-fat diet-induced obese mice compared with the normal controls [15]. Furthermore, we found that there is good correlation of the concentrations of glycerol (*r* = 0.981, *P* = 5.47 × 10^−4^) and FFA (*r* = 0.975, *P* = 1.02 × 10^−5^) between the cell lysates and the culture medium (Figure 5), which provides a more comprehensive index for lipid metabolism.

## 3. Experimental Section

### 3.1. Primary Culture of Porcine S-V Cells

Seven-day-old Taihu piglets were killed by exsanguination in a manner approved by the Sicuan Agricultural University Institutional Animal Care and Use Committee. Stromal-vascular cells (S-V cells) were isolated according to published protocols [16,17] with the following modifications: Subcutaneous adipose tissue was collected from the neck and back of the piglets and rinsed with serum-free medium (DMEM/F-12 medium supplemented with 15 mM NaHCO_3_, 100 U penicillin/mL and streptomycin). The tissue mass was cut with scissors into fine pieces and digested with type-IV collagenase (DMEM/F-12 + 20 g/L BSA+1 g/L IV type collagenase) at 37 °C in a shaking water bath for 1 h, Then, DMEM/F-12 medium containing 10% fetal bovine serum (FBS) was added to stop digestion. The solution was filtered through sterile nylon mesh (100 μm pore size) to remove undigested tissues. The filtrate was centrifuged at 800 × g for 8 min to separate the floating adipocyte cells from the pellet of S-V cells. The S-V cells were then incubated with erythrocyte lysis buffer (0.154 M NH_4_Cl, 10 mM KHCO_3_, 0.1 mM EDTA) at room temperature for 10 min [18], followed by centrifugation at 500 × g for 5 min. The S-V cell pellet was washed with DMEM/F-12, centrifuged, and resuspended in plating medium (20% FBS, DMEM/F-12). Finally, cells were seeded in culture plates at a density of 5 × 10^4^ cells/cm^2^ and cultured at 37 °C in a humidified atmosphere containing 5% CO_2_. The medium was changed every second day.

For cell growth curve studies, preadipocytes were seeded in 96-well culture plates at a density of 10^4^/cm^2^, and then 100 μL DMEM/F-12 medium containing 10% FBS was added to each well. At various points, medium was removed, we added 20 μL of the reagent into each well of the 96 well assay plate containing the cells in 100 μL of culture medium, and then cultured the cells for 1.5 h at 37 °C in a humidified, 5% CO_2_ atmosphere. Afterwards, record the absorbance at 490 nm using a 96 well plate reader. The viable cell number is proportional to the absorbance.

### 3.2. Differentiation of Adipocytes

Cultured preadipocytes were maintained in plating medium until confluence. Then, to induce differentiation, the cultures were exposed to medium containing 10% FBS, 5 μg/mL insulin, 0.5 mM 3-isobutyl-1-methylxanthine, 0.25 M dexamethasone and antibiotics in DMEM/F-12 for 48 h. This medium was then replaced with a lipid-filling medium (10% FBS, 5 μg/mL insulin, DMEM/F-12) to permit the adipocytes to accumulate TG. To qualitatively assess cell differentiation by microscopy, fully differentiated porcine adipocytes were fixed in 10 % formalin and stained with 0.3% Oil Red O (ORO) for lipid. In addition, the mRNA abundance of nine adipocyte-specific marker genes in porcine adipocyte was determined using a quantitative PCR (q-PCR) approach. The primers that were used for q-PCR are listed in Table 1.

### 3.3. Transient Transfection with the Synthetic miRNA Mimics and Inhibitors

Mimic and inhibitor oligonucleotides of ssc-miR-143-5p and ssc-miR-27a-3p (miRBase IDs: MIMAT0002148 and MIMAT0017374 respectively) and a negative control (with no sequence similarities to any reported porcine gene sequence) were synthesized by Ribobio (China). The 5-FAM fluorescence-labeled delivery control (Ribobio) was used to measure the transfection efficiency of porcine adipocytes. Mature adipocytes could be used for transfection in day 10, prior to miRNA transfection, the cell culture medium was replaced by a Serum-Reduced Medium, Opti-MEM I (Invitrogen, Carlsbad, CA, USA). The transfection was carried out using the lipid carrier Lipofectamine 2000 (Invitrogen, Carlsbad, CA, USA) according to the manufacturer’s instructions. Oligonucleotides (100 nM) prepared with lipid carrier (2:1, v/v) were subjected to the transfection in a 100 μL of Opti-MEM medium for each well of the standard 96-well plates. The adipocytes were incubated with the oligonucleotides/Lipofectamine complex for 6 h and then switching the Opti-MEM I medium to a growth medium. The cells were cultured for 3 days at 37 °C, and then subjected to the following analyses. In order to determine the optimal transfection conditions, the adipocyte viability was assessed at 24 h after transfection [19] using the MTT method.

### 3.4. Lipid Metabolism Assay

The concentrations of TGs in the lysates of adipocytes, and of glycerol and FFAs in both the lysates of adipocytes and in the culture medium were measured using the commercial kits (Applygen Technologies, Beijing, China) according to the manufacturer’s instructions. The concentrations of the TGs, glycerol and FFAs were normalized to the protein content (μM/mg protein) using a bicinchoninic acid (BCA) assay kit (Pierce Chemical, Rockford, IL, USA).

### 3.5. Statistical Analysis

The statistical significance of variations between the two variables was calculated by Student’s *t*-test (two-tailed). The Pearson’s correlation was used to determine the correlation of the concentrations of glycerol and FFA between the cell lysates and the culture medium. Values are mean ± S.D. (*n* = 3).

## 4. Conclusions

In summary, our results confirmed that miR-27a and miR-143 play important roles in porcine adipocyte lipid metabolism both in human and rodents. Simply put, miR-27a could accelerate the hydrolysis of TG, while miR-143 could promote TG synthesis. In addition, we demonstrated that there was good correlation between the indicators of adipolysis in cell lysates and in the culture medium.

## Figures and Tables

**Table 1:** List of gene specific primers used for q-PCR.

Gene Symbol		GenBank ID	Primer Sequence (5′→3′)	Product Size (bp)
Lipogenic Transcription	*C/EBP* γ	DQ450678	F: GTCCAAACCAACCGCACAT R: GAAACAACCCCGTAGGAACAT	262
	*PPAR* γ	AB097926	F: GTTGATTTCTCCAGCATTTCCA R: GGCTCTTCGTGAGGTTTGTTG	188
	*SREBP-1*	AY496867	F: AAGCGGACGGCTCACAATG R: CGCAAGACGGCGGATTTAT	122
Fatty Acid Synthase	*GPDH*	U97255	F: TGTGACTGGAAAACGGTGGC R: CGTGCAGGCATACTCCTTAATT	103
	*ACC*	AF175308	F: AAAGAGGTTCCAGGCACAGTC R: CGGTGGGAGGTATGCTTGAGT	118
	*SCD-1*	AY487829	F: GAATGACGTTTATGAATGGGC R: CAGCTTCTCGGCTTTTAGGT	190
Fatty Acid Transportation	*LPL*	NM_214286	F: TGCCCTGTAACTTCTACCCCA R: GGCAAGTGTCCTCAACTGTGTC	208
	*FAT/CD36*	DQ192230	F: GGGTTAAAACAGGCACGGAA R: TGGCACCATTGGGCTGTAGG	201
	*aP2*	DQ450677	F: GACAGGAAAGTCAAGAGCACCA R: CGGGACAATACATCCAACAGAGT	228
Housekeeping Gene	ACTB*	DQ178122	F: TCTGGCACCACACCTTCT R: TGATCTGGGTCATCTTCTCAC	114
	TBP*	DQ178129	F: GATGGACGTTCGGTTTAGG R: AGCAGCACAGTACGAGCAA	124
	TOP2B*	AF222921	F: AACTGGATGATGCTAATGATGCT R: TGGAAAAACTCCGTATCTGTCTC	137

Abbreviations: *C/EBP*γ, CCAAT element-binding protein-beta; *PPAR*γ, peroxisome proliferator-activated receptor-gamma; *SREBP-1*, sterol regulatory element-binding protein-1; *GPDH*, Glycerol-3-phosphate dehydrogenase; *ACC*, acetyl-CoA carboxylase; *SCD-1*, stearoyl-CoA desaturase-1; *LPL*, lipoprotein lipase; *FAT/CD36*, fatty acid translocase; *aP2*, adipocyte-specific fatty acid binding protein;

* ACTB (beta-actin), TBP (TATA box binding protein) and TOP2B (topoisomerase II beta) were the endogenous control genes.
